# The economic impact of chronic fatigue syndrome in Georgia: direct and indirect costs

**DOI:** 10.1186/1478-7547-9-1

**Published:** 2011-01-21

**Authors:** Jin-Mann S Lin, Stephen C Resch, Dana J Brimmer, Andrew Johnson, Stephen Kennedy, Nancy Burstein, Carol J Simon

**Affiliations:** 1 Chronic Viral Diseases Branch, Mail Stop A-15, Centers for Disease Control and Prevention, 1600 Clifton Road NE, Atlanta, GA 30333, USA; 2 Abt Associates, Cambridge MA, USA; 3 Center for Health Decision Science, Harvard School of Public Health, 718 Huntington Ave, Boston MA 02115, USA; 4 The Lewin Group, Eden Prairie, MN, USA

## Abstract

**Background:**

Chronic fatigue syndrome (CFS) is a debilitating chronic illness affecting at least 4 million people in the United States. Understanding its cost improves decisions regarding resource allocation that may be directed towards treatment and cure, and guides the evaluation of clinical and community interventions designed to reduce the burden of disease.

**Methods:**

This research estimated direct and indirect costs of CFS and the impact on educational attainment using a population-based, case-control study between September 2004 and July 2005, Georgia, USA. Participants completed a clinical evaluation to confirm CFS, identify other illnesses, and report on socioeconomic factors. We estimated the effect of CFS on direct medical costs (inpatient hospitalizations, provider visits, prescription medication spending, other medical supplies and services) and loss in productivity (employment and earnings) with a stratified sample (n = 500) from metropolitan, urban, and rural Georgia. We adjusted medical costs and earnings for confounders (age, sex, race/ethnicity, education, and geographic strata) using econometric models and weighted estimates to reflect response-rate adjusted sampling rates.

**Results:**

Individuals with CFS had mean annual direct medical costs of $5,683. After adjusting for confounding factors, CFS accounted for $3,286 of these costs (p < 0.01), which were driven by increased provider visits and prescription medication use. Nearly one-quarter of these expenses were paid directly out-of pocket by those with CFS. Individuals with CFS reported mean annual household income of $23,076. After adjustment, CFS accounted for $8,554 annually in lost household earnings (p < 0.01). Lower educational attainment accounted for 19% of the reduction in earnings associated with CFS.

**Conclusions:**

Study results indicate that chronic fatigue syndrome may lead to substantial increases in healthcare costs and decreases in individual earnings. Studies have estimated up to 2.5% of non-elderly adults may suffer from CFS. In Georgia, a state with roughly 5.5 million people age 18-59, illness could account for $452 million in total healthcare expenditures and $1.2 billion of lost productivity.

## Background

Approximate 4 million U.S. adults suffer from chronic fatigue syndrome (CFS) [1]. Afflicted individuals endure chronic, incapacitating physical and mental fatigue that is not relieved by rest. Fatigue is exacerbated by physical or mental exertion and is accompanied by impaired memory and concentration, unrefreshing sleep, muscle and joint pain, and other defining symptoms [2-4]. The pathophysiology of CFS remains inchoate; there are no diagnostic clinical signs or laboratory markers.

CFS patients, their families, employers and society bear significant costs associated with the illness. The symptoms characterizing CFS are common to many illnesses. Hence, diagnosis is complex and requires exclusion of medical and psychiatric conditions that present similarly and requiring extensive diagnostic testing and clinical assessment [2-5].

There is no known cure. CFS management aims to relieve symptoms. Treatment is long-term and its associated costs can stretch for years or a lifetime. Patients, frustrated with lack of acceptable recovery often consult several providers and self-medicate their illness [6-9].

Finally, CFS can limit patients' ability to work and participate in other life activities. Illness frequently leads to withdrawal from the labor force, absenteeism or presenteeism. As patients earnings fall, burdens on employers and family members rise as afflicted individuals cease to engage in the home and the workplace [7-9].

International studies have documented the economic costs of CFS. An Australian study examined the healthcare utilization cost and the indirect costs incurred through cessation or reduction in employment during 1988 [7]. The total annual healthcare cost for one CFS patient in Australia amounts to approximately $A2,000, of which $A1,268 was directed to the government. The mean income forgone by CFS patients in Australia was $A7,500 per year, of which $A1,700 was in tax revenue loss for the government. Overall, the economic impact of CFS to the Australian government and the community is approximately $A9,500 per patient. A Canadian study examined direct health care costs associated with fibromyalgia (FM) within a representative community sample [10]. The annual direct medical cost in 1994 for FM to affected individuals was approximately $C2,275. McCrone et al assessed the economic cost of chronic fatigue and chronic fatigue syndrome in UK primary care between January 1999 and June 2001 [9]. The mean total cost of services and lost employment across the sample was £1906 for the 3-month period with formal services accounting for 9.3% of this figure. Over 90% of the cost was accounted for by care provided by friends and family members and by lost employment. Patients with dependants had significantly higher costs than those with none and costs were also significantly higher for greater levels of functional impairment.

Two published studies have estimated the economic burden of CFS in the U.S [11,12]. The first [11] was based on the 1997/1998 baseline data from a longitudinal population-based study on CFS between 1997 and 2003 in Wichita, Kansas. Data were collected by telephone interview from a sample that included 43 persons with CFS, 3,485 suffering fatiguing illness that was not CFS, and 3,634 non-fatigued controls. Persons with CFS had 37% lower household productivity and 54% lower labor force productivity than comparable controls, resulting in annual productivity losses of between $12,000 and $28,000. A second study [12] focused on the direct medical costs associated with CFS. Utilizing archival health utilization data from an epidemiological study of CFS in Chicago, Illinois conducted between 1995 and 1998, the study computed incremental healthcare costs for 21 persons with CFS as compared to 15 healthy controls. The authors estimated that persons with CFS spent $2,342 more annually on direct medical costs than healthy individuals did.

The current study extends the prior work in four important dimensions. First, the study employs validated standardized criteria [13] that are presently recommended [5], including medical and psychiatric evaluations, to diagnose CFS. Second, it draws on a larger sample of CFS and non-fatigued individuals than available in prior work, permitting a richer and more precise analysis of healthcare and employment costs related to CFS. Third, the study uncovers an important pathway to the "productivity costs" related to CFS. CFS can forestall educational attainment, and hence illness places afflicted individuals on a lower trajectory of lifetime earnings. Finally, the study is more comprehensive than prior work, directly collecting data from patients on detailed direct medical expenditures, employment and earnings. This permits us to examine healthcare use and employment effects from the same sample, and track their relationship.

## Methods

The objective of this study is to quantify the incremental direct medical costs and indirect costs (productivity loss) associated with CFS. Incremental costs of CFS are measured relative to a non-fatigued sample of individuals, adjusting for potentially confounding characteristics. Analyses also examine incremental costs associated with "insufficient fatigue," an illness classification which is defined as intermediate to CFS, but is not known to be a precondition to CFS.

### Human Subjects

This study adhered to human experimentation guidelines of the U.S. Department of Health and Human Services, was approved by the Centers for Disease Control and Prevention (CDC) Human Subjects Review Board, and all participants gave informed consent.

### Study Sample

The study population, clinical and psychiatric evaluations have been described in previously published work [1]. Briefly, between September 2004 and July 2005 we initially sampled non-elderly individuals from metropolitan, urban, and rural communities around Atlanta and Macon, Georgia [1]. A random-digit-dialing telephone screening interview initially classified respondents either as "well" or as having "symptoms of CFS." A follow-up, detailed telephone interview was administered to all individuals with symptoms and also to a probability sample of those without symptoms. Based on the detailed interview, those meeting criteria of the 1994 CFS case definition [2] were classified as "CFS-like." Other respondents were classified as either "unwell" (but not CFS-like) or "well." All "CFS-like" individuals were recruited for a one-day clinical evaluation. A random sample of those who were unwell but not CFS-like, and a set of matched well people were also recruited for clinical evaluation, yielding a clinical evaluation sample of 783 individuals.

The clinical evaluation involved medical and psychiatric evaluations to identify exclusionary conditions. Participants were also queried on healthcare and drug utilization, out-of-pocket spending, health insurance, education, employment, earnings, access to care and socioeconomic characteristics using validated questions taken from the Medical Expenditures Panel Survey (MEPS) [14] and the Current Population Survey [15].

We identified medical or psychiatric conditions exclusionary for CFS in 280 (36%) of 783 clinic participants. The four most common exclusions were thyroid disease (24%), anemia (18%), uncontrolled diabetes (14%), and autoimmune disease (11%). In addition, 2 individuals' status was undetermined because of incomplete lab and another one did not complete healthcare utilization data.

The clinical evaluation permitted us to classify the remaining 500 non-excluded participants into one of three groups: CFS, insufficiently fatigued, or non-fatigued. Participants were classified as CFS if they met the criteria of the 1994 case definition [2] as applied following recommendations of the International CFS Study Group [5], as currently utilized by CDC [13]. Those who met at least one but not all criteria for CFS were classified as "unexplained chronic illness with insufficient symptoms or fatigue for CFS" (termed ISF). Those who met none of the criteria were classified as "non-fatigued, well" (termed NF). Of the 500, non-excluded individuals, we classified 112 as CFS, 264 as ISF, and 124 as NF. All analysis reported below were based on the sample of 500 individuals. Table 1 summarizes their characteristics.

**Table 1:** Characteristics of Study Population by CFS-related Health Status Classification^a^

Sample	ALL	CFS	ISF	NF
Unweighted Number of Individuals	500	112	264	124
Estimated prevalence in sampled population (%)	100	4.9	52.1	43.0

**Sex**	**Weighted Column Percent**			
	**ALL**	**CFS**	**ISF**	**NF**

Female	44.5	56.5	47.9	39.1
Male	55.5	43.5	52.2	60.9

**Race**	**Weighted Column Percent**			
	**ALL**	**CFS**	**ISF**	**NF**

White, Non-Hispanic	65.4	65.2	55.8*	77.1
Black, Non-Hispanic	27.8	24.6	33.5*	21.2
Other	6.8	10.3	10.7*	1.7

**Age Group**	**Weighted Column Percent**			
	**ALL**	**CFS**	**ISF**	**NF**

18 to 29	38.0	11.3	43.6	34.3
30 to 39	23.9	26.6	19.9	28.4
40 to 49	18.2	28.6	19.9	15.0
50 to 59	19.9	33.6	16.6	22.3

**Age**	**Weighted Years**			
	**ALL**	**CFS**	**ISF**	**NF**

Mean	35.8	43.0**	34.6	36.4
Standard Deviation	12.58	5.15	12.8	15.9

**Marital Status**	**Weighted Column Percent**			
	**ALL**	**CFS**	**ISF**	**NF**

Married	54.1	62.5	55.2	51.8
Not Married	45.9	37.5	44.8	48.2

**Education**	**Weighted Column Percent**			
	**ALL**	**CFS**	**ISF**	**NF**

College degree or higher	31.0	26.8	31.3	31.2
Less than a college degree	69.0	73.2	68.7	68.8

**Local Market Conditions (Sampling Stratum)**				
	**ALL**	**CFS**	**ISF**	**NF**

Metro	19.6	20.3	18.9	20.4
Urban	32.6	29.4	44.8	18.2
Rural	47.8	50.4	36.3	61.5

**Health Insurance**	**Weighted Column Percent**			
	**ALL**	**CFS**	**ISF**	**NF**

Any Private	74.6	77.2	66.7	85.5
Public Only	4.5	8.6	1.4	8.2
None	20.9	14.2	31.9	6.3

**Employment/Earnings**	**Weighted Percent and Weighted Means**			
	**ALL**	**CFS**	**ISF**	**NF**

Employment Status: % Who Worked at all in last 4 weeks	78	71	65	95
Annual Earnings†	$28,188	$23,076	$23,856	$33,888

^a ^All 500 participants reported gender, race, age, and stratum, 463 reported health insurance status.* ISF different than NF (2-sided T-test, alpha = 0.05), ** CFS different than non-CFS (ISF+NF) (2-sided T-test, alpha = 0.05). All estimates weighted to reflect sampling probabilities. †Computed by multiplying reported 4-week earnings by 13.

### Sampling Weights

The clinical evaluation sample overrepresented people with CFS; so, we constructed sampling weights for our estimates. We utilized sampling weights in statistical analyses, so that standard errors of our estimates included uncertainty stemming from the complex sample design.

CFS and ISF identified at the clinical evaluation were from the telephone screening samples of CFS-like and chronically unwell subjects. Initially, we sampled CFS-like and chronically unwell subjects from the Georgia population with a known probability. Sampling weights incorporated a further adjustment for clinical evaluation nonresponse and reflected the probability of selecting chronically unwell people for clinical-evaluation. Because "well" subjects were selected for clinical evaluation based on matching to CFS-like, we estimated their sampling rates based on the response-rate adjusted sampling rates for the underlying sample of completed telephone interviews. Our estimation strategy for weights and the statistical properties of the weighted analyses are found in Additional file 1. Briefly, we estimated pseudo-weights, by stratum, for the matched sub-sample based on observed demographic characteristics and the known sampling weights for similar individuals in the CFS-like sample. We weighted the 3 sampling strata in proportion to their size.

### Data

Analyses required four types of data:

• Healthcare Utilization: Utilization data were used to calculate direct healthcare expenditures. Data were collected from surveys administered as part of the clinical evaluations. Survey questions queried inpatient hospitalizations, outpatient provider visits, prescription drugs, non-prescription drugs.

• Healthcare prices: Prices were required to convert utilization numbers to costs. Prices were not collected in the study, but were obtained from nationally representative surveys and data sources, including the Medical Expenditure Panel Survey (MEPS) and the Pharmaceutical Red Book, detailed below.

• Employment and earnings histories: Data were obtained from surveys conducted during the clinical evaluation using questions modeled on the Current Population Survey and MEPS.

• Demographic Information: Data on individual's socioeconomic characteristics, household structure and education were collected from surveys administered during the clinical evaluation.

#### Healthcare utilization

Participants reported inpatient hospitalizations in the past year, outpatient encounters with healthcare providers in the past 6 months, prescription medications filled in the last 4 weeks, and other medical supplies and services obtained in the past 4 weeks. For hospitalizations, participants indicated length of stay (LOS) in the hospital and occurrence of surgical procedures. For outpatient encounters, respondents indicated number of encounters with various providers (medical doctor or osteopath, nurse or physician's assistant, mental health, alternative or complementary medicine). For prescription medications, the names of up to 14 prescription medications purchased in the past 4 weeks were reported, and the amount spent out-of-pocket for each. Participants did not itemize non-prescription medications, but reported the amount they spent on all non-prescription drugs purchased in the last 4 weeks. For other supplies/services utilization they provided a description of the purchase.

#### Prices

We did not ask participants to report expenses for hospitalizations, office visits, or prescription medication expenses. To obtain estimates of expenses, we employed the data from the 2005 Medical Expenditure Panel Survey [14] to compute average per-day costs for medical and surgical inpatient hospitalizations and for encounters with healthcare providers; accounting for age, region, population density, and health insurance. We estimated prescription drug costs as the average wholesale prices from the 2007 Drug Topics Red Book for reported prescription medications [16]. We assumed that generic equivalents were purchased whenever available. The total cost of non-prescription medications and other supplies and services were assumed to be the reported out-of-pocket expenditures for these items.

All utilization, expenditure, and earnings data were annualized, assuming that average per-capita expenditures and earnings for the population occurred at a constant rate over the year. Dollar estimates were converted to 2005 price levels.

#### Employment and Earnings

The detailed telephone interview and the clinical evaluation included questions that are patterned after the U.S. Current Population Survey [15] to measure current employment, type of employment, full or part time status, hours worked over the past 4 weeks, individual earnings, household earnings, employment history/experience and educational attainment

#### Socio-demographic characteristics

Other modules of the clinical evaluation included additional information on individual socio-demographic characteristics, including age, race, sex, marital status, household composition, insurance status and metro/urban/rural location. We chose the potential socio-demographic confounders on the basis of their association with healthcare costs and CFS. CFS has been shown to be associated with socio-demographics such as sex or gender [1,3,17], age [3,13], race or ethnicity [3,18].

### Analysis

#### Outcomes of Interest

The economic burden to society attributable to CFS can be separated into: (1) direct medical costs associated with diagnosing and treating CFS and, (2) indirect costs stemming from lost productivity by persons with CFS.

We measured direct cost as total annual healthcare expenditures disaggregated into 5 utilization categories: 1) inpatient hospitalization; 2) provider encounters; 3) prescription medications; 4) over-the-counter (OTC) medications; and 5) other healthcare costs. For each category we analyzed total costs and the out-of-pocket portion of costs borne by patients. Separate analyses of the respective categories permitted us to observe differences in patterns of utilization between fatigued and non-fatigued individuals, contributing to a better understanding of cost drivers.

We measured indirect, productivity costs based on participants' self-reported earnings in the past 4 weeks. To better understand the underlying determinants of earnings, we separately estimated the effect of CFS on participants' labor force participation and educational attainment.

#### Contrasts

We constructed two alternative contrasts to measure the incremental burden of fatiguing illness:

1. CFS compared to NF,

2. ISF compared to NF.

Comparisons between the CFS and NF groups may be thought as capturing the effects of alleviating all fatiguing symptoms. Comparisons between ISF and NF capture the costs associated with an intermediate state that meets some but not all the fatiguing criteria associated with CFS.

#### Estimation Strategy

We could not directly observe the counter-factual healthcare costs or employment outcomes that would have occurred if persons with fatiguing illness had not been ill. Therefore, we used econometric models to compare fatigued individuals to those classified NF. The incremental healthcare costs (or earnings) of persons with CFS were estimated as the difference in costs between CFS and NF groups, adjusted for other factors that may affect healthcare costs (or earnings) but are also associated with fatiguing illness. Similarly, we estimated the incremental cost of ISF as the adjusted difference in expenditures and earnings between the ISF and NF groups. We used indicator variables in our econometric models to capture CFS and ISF effects, with NF serving as referent group. Covariates included the socioeconomic variables described in Table 1.

Weighted analyses were performed to account for the probability of being selected to participate in the clinical evaluation. For continuous outcome measures (e.g. total healthcare expenditures and total earnings), we incorporated the sampling weights in models: ordinary least squares linear regression (Linear OLS) on untransformed costs (Linear OLS), generalized linear model (GLM) with appropriate distribution (gamma or other distributions determined by Modified Part Test on distribution family choice) and a log link function, and two-part model (TPM) with the first part of logit model and the second part of GLM. Models were selected based on goodness of fit/deviance, and information criteria such as -2 Log-Likelihood, AIC, BIC, etc. For dichotomous outcome measures (e.g. indicators for working in any of the past 4 weeks) we estimated a logistic regression. The statistical significance of the estimated effects, odds-ratios and model coefficients were reported as p-values.

#### Effects of CFS on educational attainment and earnings

The estimated effect of CFS on worker productivity and employment is based on a "human capital" framework, in which an individual's earnings are influenced by their education and experience. However, many participants developed CFS during prime schooling years, and prior research has shown that educational attainment is not independent of many illnesses. Including observed educational attainment as a covariate, could underestimate the overall effect of CFS on earnings, if developing CFS at a young age reduced a person's ability to attend or perform well in school.

Isolating the effects of CFS on earnings required that we develop a measure of educational attainment abstracted from the potential effects of fatigue on observed education. We hypothesized that illness most likely interfered with education for individuals who reported onset of CFS prior to age 25. Taking the sample of individuals who suffered CFS onset *after age 25 *we estimated an educational attainment model based on location and socio-demographic characteristics. We then applied the education-attainment models to the early-onset CFS sample to forecast the education that these individuals might have achieved had illness developed later.

Since including an unadjusted 'educational attainment' covariate in our regression analysis would lead to an underestimate of the effect of CFS, we imputed educational attainment for those with CFS based on their predicted education attainment *if onset had CFS occurred after age 25*; the imputation process excludes individuals with CFS who are younger than 25, some of whom might still attain a BA before their 25th birthday. Restricting our sample to those individual with CFS who are 25 and older, we estimated the following regression:

$$Log\left(\frac{\mathrm{Pr}(BA_{i}=1)}{1-\mathrm{Pr}(BA_{i}=1)}\right)=\beta_{0}+\sum_{k=1}^{K}\beta_{k}X_{ik}+\beta_{K+1}(Onset_{i})$$

where, *Pr(BA_i_*) is the probability that individual (*i*) completes college, *BA_i _*( = 1 if true, 0 otherwise)

*X_i _*is a vector of covariates for the *i*^th ^individual presumed to effect college completion (including age, sex, race/ethnicity, marital status and urban areas)

*β_k _*is the estimated effect of the *k*^th ^covariate on the outcome

*Onset_i _*is whether a person has late onset of CFS (i.e. after the age of 24), and

*β_K+1 _*is the estimated effect of late onset on the outcome, and

*β_0 _*is an intercept term

Using estimates from the above regression, we calculated the predicted value of college completion (for the entire 25+ year-old CFS population), assuming all individuals have late onset of CFS, regardless of whether CFS onset was actually early or late. This prediction was our new imputed value of education. It predicts the education individuals would have had, on average, absent CFS, effectively eliminating the indirect effect of CFS on employment and earnings through education.

Hence, CFS is expected to have two effects on earnings. The first is the contemporaneous effect of illness on employment and productivity that is observed by comparing a person's current earnings to others with like education and experience. The second cost of CFS is due to diminished educational attainment. CFS reduces an individual's ability to complete education, which in turn, places the individual on a lower trajectory for earnings over their lifetime.

## Results

### Direct Medical Costs

Table 2 summarizes annual healthcare expenditures for individuals classified as CFS, ISF, and NF. For comparison, we have also reported healthcare expenditures for the U.S. population age 18 to 59, calculated from the 2005 MEP Survey. Healthcare expenditures for the Georgia sample ($2,767) were, on average, not greatly different from those reported by similarly aged US adults ($2,963), as estimated in the 2005 MEP Survey.

**Table 2:** Mean Annual Healthcare Expenditures of the Sampled Population, with Comparisons to the Non-elderly US Population (in US Dollars, $)

*Unadjusted sample means, weighted to account for sample design*				
	***Healthcare Expenditure Category***			

**Sample**	**Inpatient Hospitalization, mean costs** **($2005)**	**Ambulatory Provider visits** **mean costs** **($2005)**	**Rx Medications,** **mean costs** **($2005)**	**OTC Medications and other healthcare costs;** **mean costs** **($2005)**

CFS	902	3,217	1,347	217
ISF	484	1,702	648	134
NF	303	1,406	294	93
Study Sample	435	1,665	546	122
US non-elderly adult Population*	801	1,474	624	64

*US population estimates based on 2005 MEPS, age 18-59; The MEPS estimate of 'other' costs includes OTC medications. Rx = Prescription; OTC = non-prescription ('over-the-counter').

Individuals with CFS reported significantly higher healthcare expenditures, in total and separately in all utilization categories. Mean expenditures for persons with CFS were $5,683, almost double the mean costs reported by the ISF sample ($2,968) and 170% higher than NF ($2,096).

Table 3 summarizes the adjusted results from Linear OLS, Log OLS, GLM, and TPM for each category of healthcare expenditure. The incremental total expenditures attributed to CFS estimated from the GLM gamma with a log link function ($3,286) was less than 10% relative difference from untransformed Linear OLS ($3,085) and TPM ($3,618). The -2 log-likelihood for the TPM on total expenditures was 8214.72, smaller than that for the GLM (8647.42). However, AIC penalized the two-part model (13-54 folds) and Linear OLS (460-2373 folds) for its additional parameters for each category of healthcare expenditure. Thus, we selected the results from GLM for inferences in this paper.

**Table 3:** Estimated Regression-adjusted Effect of CFS and ISF on Annual Healthcare Expenditures (in US Dollars, $)

*All effects estimated relative to not-fatigued (NF) population, (Standard Errors in Parentheses)*						
	**Linear Ordinary Least Squares (OLS) for Untransformed Cost (y)**					

	**Total Expenditures** **($2005)**	**Inpatient Hospital** **Expenditures** **($2005)**	**Ambulatory Provider Visits** **($2005)**	**Rx Medications** **($2005)**	**OTC Medications** **($2005)**	**Other Health Costs** **($2005)**

Incremental Expenditures attributed to CFS	3084.95*** (799.09)	476.11 (437.06)	1590.70*** (598.98)	919.18*** (293.37)	111.99*** (36.98)	-13.03 (12.43)
Incremental Expenditures Attributed to ISF	1119.75** (476.46)	262.89 (254.04)	364.41 (272.99)	448.54*** (142.68)	31.81 (26.80)	12.09 (11.35)
AIC	8303.74	7815.93	7654.30	6969.76	5196.03	6361.90
BIC	8306.15	7818.34	7656.70	6972.17	5198.44	6364.30

	Generalized Linear Model for Untransformed Cost ((y+1)) ^a^					

Incremental Expenditures attributed to CFS	3285.54*** (1026.50)	519.95 (582.17)	1343.25** (587.55)	1241.13* (694.19)	204.01* (111.16)	-1.83 (1.28)
Incremental Expenditures Attributed to ISF	1058.27*** (352.68)	71.61 (122.50)	384.73 (250.49)	317.41** (131.90)	46.83 (30.66)	0.82 (1.37)
AIC	17.33	11.12	16.62	12.54	10.37	2.68
BIC	-2045.41	1178.77	-1739.82	-205.99	-925.17	-2767.13

	Two-Part Model (TPM) for Untransformed Cost (y)^b^					

Incremental Expenditures Attributed to CFS	3618.21*** (1285.90)	602.70 (907.17)	1622.69*** (553.52)	1192.25*** (391.32)	189.13*** (54.14)	-16.30 (21.12)
Incremental Expenditures Attributed to ISF	1151.63 (751.45)	128.18 (577.98)	416.12 (322.03)	473.56*** (180.19)	63.30** (25.14)	30.11 (37.49)
AIC	292.26	150.16	463.65	548.09	564.80	86.65
BIC	-2113.67	1359.96	-1250.64	371.70	-328.59	-1313.92

* p < 0.1, ** p < 0.05, ***p < 0.01.

^# ^Full GLM results in Additional file 2, Table S2.

AIC: Akaike's information criterion; BIC: Schwarz's information criterion.

^a ^Generalized Linear Model with Gamma distribution and the log link function for all the categories of healthcare expenditures except for "Other Health Costs" with inverse Gaussian distribution.

^b ^Two-part model: the first part using a logistic regression for binary indicator (healthcare users vs. no-users) and the second part using GLM for positive healthcare costs.

Paralleling prior research, age, sex, and race/ethnicity contributed significantly to healthcare expenditures in the GLM models, reported in full (Additional file 2, Table S2). Adjusting for covariates, those with CFS spent $3,286 more annually compared to NF (p = 0.001). Similarly, those classified as ISF spent $1,058, more annually than NF (p = 0.003). Adjusted estimates were slightly greater than the unadjusted estimates presented in Table 2, above.

Examining the pattern of expenditures helps us understand how individuals with fatiguing illness interact with the healthcare system for prevention, ambulatory care and treatment of acute conditions requiring hospitalizations. Provider visits accounted for the largest share (41%) of incremental CFS costs. Compared to NF, individuals with CFS diagnoses annually incurred an additional $1,343 (p = 0.022) for costs related to ambulatory healthcare visits (physician, dentist, nurse practitioner, therapist, chiropractor, etc). Prescription drugs were the next largest driver (38%) of additional healthcare costs for the CFS sample. Individuals with CFS spent $1,241 (p = 0.074) more on prescription drugs annually than comparable non-fatigued patients. CFS patients also spent more on over-the-counter medications ($204, p = 0.066). Hospitalization for the CFS sample is an infrequent event. While those with CFS exhibit slightly higher expenses when hospitalized, their hospitalization rate was not significantly greater than the non-fatigued sample. Hence, inpatient costs were not statistically significantly raised for those with CFS.

ISF patients had healthcare utilization and cost profiles that fell between the NF samples and the CFS samples: ISF raised healthcare cost by over $1,058 annually (p = 0.01). The largest portion (30%) of the incremental cost of ISF was attributed to prescription medications, which increased by $317 per year relative to controls (p = 0.016). Costs for ambulatory care were also higher in the ISF sample, but not statistically significantly so.

Out of pocket costs are often the best measure of the direct financial burden faced by patients. CFS raised patients' total out-of-pocket (OOP) expenditures by $947 per year relative to the NF sample (p = 0.003, GLM results in Table 4). Increases in prescription medication costs, provider visit costs, and over-the-counter medication purchases represented 83%, 29% and 22% (respectively) annual out-of-pocket cost burden attributed to CFS. We estimated out-of-pocket costs for the ISF sample to be $482 higher than NF (p < 0.001). As with total expenditures, prescription medications were the largest portion of the incremental out-of-pocket costs for ISF.

**Table 4:** Estimated regression-adjusted effect of CFS and ISF on annual out-of-pocket healthcare expenditures (in US Dollars, $)

*All effects estimated relative to not-fatigued population, (Standard Errors in Parentheses)*						
	**Linear Ordinary Least Squares (OLS) for Untransformed Cost (y)**					

	**Total Expenditures** **($2005)**	**Inpatient Hospital** **Expenditures** **($2005)**	**Ambulatory Provider Visits** **($2005)**	**Rx Medications** **($2005)**	**OTC Medications** **($2005)**	**Other Health Costs** **($2005)**

Incremental Expenditures attributed to CFS	716.47*** (237.12)	22.50 (18.05)	234.35*** (84.29)	360.66** (171.28)	111.99*** (36.98)	-13.03 (12.43)
Incremental Expenditures Attributed to ISF	526.88*** (189.66)	8.74 (7.18)	95.15** (38.31)	379.09** (172.37)	31.81 (26.80)	12.09 (11.35)
AIC	7164.63	4544.74	5634.06	6906.44	5196.03	6361.90
BIC	7167.03	4547.15	5636.46	6980.84	5198.44	6364.30

	Generalized Linear Model for Untransformed Cost ((y+1)) ^a^					

Incremental Expenditures attributed to CFS	946.73*** (323.22)	16.61 (14.77)	270.26** (106.17)	783.05 (641.38)	204.01* (111.16)	-1.83 (1.28)
Incremental Expenditures Attributed to ISF	481.50*** (111.62)	-0.88 (1.54)	119.13*** (39.69)	201.83** (102.30)	46.83 (30.66)	0.82 (1.37)
AIC	14.46	3.02	12.85	11.64	10.37	2.68
BIC	-2207.85	-2754.06	-2039.43	-310.92	-925.17	-2767.13

	Two-Part Model (TPM) for Untransformed Cost (y) ^b^					

Incremental Expenditures attributed to CFS	950.18** (372.49)	-51.88 (145.88)	270.08** (111.50)	1024.33* (537.26)	21.36 (51.29)	-480.13*** (46.27)
Incremental Expenditures Attributed to ISF	480.91*** (117.42)	-165.87 (138.01)	118.28*** (38.35)	447.29** (185.36)	25.12 (44.77)	-322.10** (146.36)
AIC	289.52	144.74	459.89	559.91	564.81	83.92
BIC	-2126.02	-1223.87	-1550.25	279.4912	-328.59	-2643.75

* p < 0.1, ** p < 0.05, ***p < 0.01.

AIC: Akaike's information criterion; BIC: Schwarz's information criterion.

^a ^Generalized Linear Model with Gamma distribution and the log link function for all the categories of healthcare expenditures except for "Inpatient Hospital Expenditures" and "Other Health Costs" with inverse Gaussian distribution.

^b ^Two-part model: the first part using a logistic regression for binary indicator (healthcare users vs. no-users) and the second part using GLM for positive healthcare costs.

### Educational attainment

A person's educational attainment is an important determinant of their earnings. Fifteen percent of the CFS sample experienced onset of CFS in their teens and early twenties, and had significantly lower educational attainment than others whose CFS symptoms developed later in life. In particular, the early-onset CFS sample had much lower rates of college and post-graduate education, with the expected adverse effect on their employment and earnings. College-educated individuals who were not fatigued earned $18,899 more annually than individuals with CFS who did not earn a college degree (p < 0.01; data statistics not shown).

To assess the impact of CFS on education, we modeled college graduation rates among CFS cases as a function of age, race, geographic location, sex, marital status, and age of CFS onset. After adjusting for demographic covariates, for individuals with known CFS at age 24 or earlier, the predicted percentage who would have finished college more than doubles (rises from 23% to 57%) when CFS onset is moved to after age 24 (p < 0.01, Table 5).

**Table 5:** Age of CFS Onset and Educational Attainment (Finishing College) in the Sampled Population

	Percentage of CFS Cases (n = 107)	Proportion Finishing College - Reported	Proportion Finishing College - predicted based on individual characteristics
CFS Onset at Age 25 or Later	53%	0.35	NA
CFS Onset at Age 24 or Earlier	15%	0.23***	0.57***
CFS Onset Age Unknown	32%	0.08***	0.34***
Combined CFS Subsample (Regardless of Timing of CFS Onset)	100%	0.24***	0.38***

Note: 4.5% (n = 5) of the 112 CFS cases were less than 25 years old. Because the education of these individuals may plausibly change up to age 25, we do not include them in our regression and do not impute values for their educational attainment. * p < 0.1, ** p < 0.05, ***p < 0.01.

Age of illness onset was unknown for 32% of the CFS sample and only 8% of those with missing onset data had graduated from college. Had these individuals experienced CFS onset at or after age 25, our model predicted that 34% would have completed college (p < 0.01).

We used our education attainment models to predict a counter-factual level of education for those with early onset CFS. Predicted education was used as a covariate in place of observed education of our earnings and employment analyses. By comparing the two model specifications - with observed versus predicted education -- we observed how much of the impact of CFS on earnings is attributable to lower educational attainment.

### Earnings and Employment

Persons with CFS earned $23,076 annually and 71% had been employed within the past 4 weeks (Table 1). These figures compare unfavorably to the NF sample who reported mean annual earnings of $33,888 and a 95% rate of employment over the 4 weeks prior to the survey.

Table 6 summarizes the estimated effects of CFS on employment and earnings over a 4-week period, after adjusting for covariates. This table provides the odds ratios (OR) of employment for the CFS (or ISF) sample relative to NF and incremental effects of CFS on earnings from the results of Linear OLS, GLM, and a two-part model (the first part using a logistic regression for binary indicator for having earnings and the second part using GLM for non-zero earnings). The results of full GLM and logistic models were reported in Additional file 2, Table S3.

**Table 6:** Impact of Fatigue on Employment and Earnings

*All effects estimated relative to not-fatigued (NF) population*, (*Standard Errors in Parentheses)*						
	**Employment**: Adjusted odds ratio (OR) of working in the prior 4 weeks relative to NF sample					
	Model with reported education			Model with imputed education		

Incremental Expenditures attributed to CFS	0.15*** (0.10)			0.12*** (0.08)		
Incremental Expenditures Attributed to ISF	0.14*** (0.08)			0.14*** (0.08)		

	**Earnings over the past 4 weeks **($), adjusted *impact measured relative to NF sample*					

	Linear OLS		GLM gamma for (y+1)		TPM	

	Model with reported education	Model with imputed education	Model with reported education	Model with imputed education	Model with reported education	Model with imputed education
Incremental Expenditures attributed to CFS	-878.24** (337.60)	-1079.47*** (347.64)	-503.11 (392.52)	-658.30** (298.83)	-266.30 (261.26)	-394.96 (255.41)
Incremental Expenditures Attributed to ISF	-408.37* (284.75)	-405.39 (283.90)	-1019.04 (654.78)	-1029.23 (665.62)	-22.37 (235.84)	-25.94 (235.60)
AIC	8397.40	8398.11	17.32	17.31	538.28	538.28
BIC	8451.49	8452.20	-1509.96	-1511.78	-1399.67	-1399.46

* p < 0.1, ** p < 0.05, ***p < 0.01.

Full model results for logistic regression on employment and GLM on earnings in Additional file 2, Table S3.

CFS and ISF each had a negative impact on earnings and on labor force participation. The model utilizing imputed educational attainment estimated a $658 reduction in 4-week earnings associated with CFS (compared to NF).

When we used reported educational attainment in the model, the effect of CFS on earnings falls to $503 per four-week period. The 24% reduction in the "CFS effect" was because the reported education specification ignored the downward bias in educational attainment that resulted from early-onset of fatigue. Because our sample was all working-age, this change was relative to a very high employment rate (95%) among well individuals. Decreasing the probability of employment by 19 percentage points in the NF group (all else being equal) would reduce average monthly earnings per person by about $330--about half of the total CFS effect on earnings (data statistics not shown in the table). Individuals with CFS were also significantly less likely than NF to have worked in the past 4 weeks (odds-ratio = 0.12, p = 0.001).

Turning to the results for the ISF sample, ISF significantly reduced the likelihood of working during the past 4 weeks. Although the point estimate on earnings suggests a negative effect as well, the estimate is not statistically significant. For examining the impact of CFS on earnings, Additional file 2, Table S4 summarizes its impact on earning controlling separately for the interaction with the reported educational attainment and the employment status. Compared to NF with BA or post graduate degree, CFS subjects without and without BA or post graduate degree had $1,320 and 611 less annual earnings, respectively. In a separate analysis controlling for the interaction of CFS and the employment status, unemployed and employed CFS subjects had $574 and 40 less annual earnings than employed NF subjects.

The combined economic burden of CFS, including both direct and indirect annualized costs, was $11,780; representing $3,226 in direct medical costs (Table 3) and $8,554 in lost earnings (Table 6). The estimate was approximately $2,015 lower using the specification that ignored the effect of illness on education.

## Discussion

We found that persons with CFS had substantially increased medical costs and reduced earnings compared to similar individuals who were not suffering from fatiguing illness. Together these economic costs totaled $11,780 annually. Lost productivity accounted for 82% of the total cost of CFS, with almost half of productivity loss attributable to lower rates of employment. The remaining loss can be attributed to reduced wage rates or reductions in hours worked per week; potentially linked to job changes that were designed to accommodate illness, absenteeism or presenteeism; but we did not measure these outcomes.

Our estimates of the direct medical cost ($3,226) and productivity loss ($8,554) associated with CFS are consistent with the findings of previous studies [11,12]. Jason et al [12] estimate direct medical costs were $2,342 and Reynolds et al [11] estimate lost earnings were $15,018. Reynolds et al [11] also estimate a mean annual lost household productivity of $5,073.

Regression adjustments were necessary to account for differences in characteristics of the CFS, ISF, and NF samples. Without adjustment, the difference in medical costs reported between the CFS and NF samples appeared larger (Table 2). CFS disproportionately affected older, white, married individuals and women. These characteristics were also significant predictors of healthcare expenditures and earnings.

The accuracy of the regression-adjusted effects we reported depends on the correctness of the regression specifications. Our regression models used an indicator variable for CFS and ISF and no interaction terms, presuming that fatigue increases medical costs but did not affect the contribution of any covariates. We tested a fully interacted model for our estimates of direct costs, but found no significantly different results.

An exception was in the models of earnings and employment. Here we found strong evidence of an interaction between CFS and a key covariate: educational attainment. Early onset of CFS materially reduced educational attainment and this in turn produced a secondary, indirect effect on earnings and on employment. Early onset CFS reduced an individual's probability of completing college by half. As college education adds significantly to lifetime earnings potential, this is a very significant finding. Accounting for the effect of early onset CFS on education materially increased estimates of lost productivity, and suggests that a significant portion of the illness economic burden may come from interrupting education or impairing an individual's ability to learn. We conducted sensitivity tests around the specification of our earnings and employment models and concluded that reported results are robust.

There is a growing body of literature that is examining the effect of illness on productivity. Increasingly researchers have uncovered evidence that illness erodes a person's accumulation of human and social capital; placing them on a lifetime trajectory of lower health and wealth [19-23] and raising the benefits of early interventions aimed at prevention, symptomatic relief, and cure [22].

ISF empirically presents as an interesting intermediate state, between NF and CFS. Clinically, the ISF sample contains individuals with some symptoms of fatigue, but symptoms are not sufficient in number or duration to satisfy the 1994 classification as CFS. Economic outcomes in general followed this clinical gradient, with ISF-associated costs generally falling between the values for CFS and NF. Observations collected over time would better enable one to explore whether ISF patients present as "mild CFS" and whether progression in fatiguing illness increases economic burden correspondingly.

Finally the direct medical costs associated with CFS and ISF are both driven by increased visits to ambulatory providers and use of prescription medications. Improvements in diagnoses and understanding "best practices" in primary care could lead to better outcomes and conservation of healthcare resources

## Strengths and Limitations

One of the strengths of this study is the use of direct medical costs with indirect costs (productivity) in a population-based clinical epidemiological study. This study employed several models commonly used to examine in the skewed healthcare cost data and investigated whether inference changes across all the models. This study has the potential to provide an important piece of evidence to inform decision making regarding resource allocation and evaluation of intervention programs.

One limitation of this study is that lack of information on dates for healthcare utilization, which prevents us from examining the time effect on healthcare utilization and costs. In our study participants reported inpatient hospitalizations in the past year, outpatient encounters with healthcare providers in the past 6 months, prescription medications filled in the last 4 weeks, and other medical supplies and services obtained in the past 4 weeks. The limitations of the survey design are 1) data limited to the state of Georgia, 2) non-institutional individuals aged 18-59, and, 3) the study was conducted in English, which prevents us from to examining non-English speaking population. Data limitations also prevented us from estimating the value of informal care giving by family and friends of people with CFS. Thus, the total societal burden of CFS could exceed our estimates.

## Conclusions

CFS attributes to significant economic costs in terms of direct medical costs and in terms of lost earnings and productivity. Similar to many other chronic illnesses, productivity loss is even larger than the costs associated with treatment. Such findings suggest that employers are major stakeholders in the search for better diagnosis and appropriate treatment since lost wages are often accompanied by absenteeism costs and health insurance costs that are borne by employers.

Given the limitations of the survey design, we cannot a-priori generalize our findings beyond the study population. However, our findings offer a benchmark for assessing the approximate magnitude of the burden of illness that results from CFS more generally in the population. In our other published work, 2.5% of adults from metropolitan, urban, and rural Georgia in our study population suffered from CFS [1]. Georgia has roughly 5.5 million people [24] age 18-59, if the similar patterns prevail CFS could account for $452 million in healthcare expenditures and $1.2 billion of lost productivity for the state.

This study is a first step in using cost-effectiveness analyses to identify the economic impact of CFS and may assist in future research by directing policy in research and education. For example, extrapolating our data to the U.S. population ages 18-59, CFS could account for as much as (or as little as) $14 billion in healthcare expenditures and $37 billion in lost productivity. However, this estimate should be interpreted with caution given that our data stem from a population-based study in Georgia. A previous CFS study^11 ^in Wichita, Kansas estimated a national economic burden of $9.1 billion per year in productivity; about 3-fold less than the current study. Estimated CFS prevalence in Kansas was about one-tenth of that observed in the Georgia study population. The differences may also reflect the increment of U.S. median household income between two study periods ($43,648 in 1997 and $46,326 in 2005). As better data become available regarding the prevalence and distribution of CFS across the U.S., it will become possible to generate more precise estimates of the burden of CFS nationally.

## Competing interests

The authors declare that they have no competing interests.

## Authors' contributions

JML, SCR, CJS, DJB and NB were involved in the conception and design. JML and CJS contributed to the primary analyses. SCR, CJS, AJ and DJB were involved in the analysis plan and interpretation of the data. SK collaborated with others in generating clinic sampling weights. SCR and CJS drafted the article. SCR, JML, DJB, and CJS were responsible for critical revision of the article for intellectual content. All authors have read and approved the final manuscript.

## Supplementary Material

Additional file 1 **Details on survey design and sampling weight**. The file includes details on the survey design and the estimation of sampling weights. Click here for file

Additional file 2 **Full model results for Tables **3 and 6. The file includes the tables for the full model results for Tables 3 and 6. The file also includes Table S4 for examining the impact of CFS one earnings from separate analyses controlling its interaction with the reported educational attainment and the employment status. Click here for file
