# Supplementary material for: The economic impact of chronic fatigue syndrome in Georgia: direct and indirect costs
Source: Cost Eff Resour Alloc. 2011 Jan 21;9:1. doi: 10.1186/1478-7547-9-1 (PMC3033815; doi:10.1186/1478-7547-9-1)
Supplement: Additional file 1 — Details on survey design and sampling weight. The file includes details on the survey design and the estimation of sampling weights. [file 1478-7547-9-1-S1.DOC]

### Additional file 1 – details on survey design and sampling weight

**Supplementary Material**

**Details on the Survey Design**

In the screening interview, we asked adult (≥ 18 years) household informants to identify *unwell* adult household members who displayed at least 1 of 8 CFS defining symptoms (fatigue, cognitive impairment, unrefreshing sleep, muscle pain, joint pain, sore throat, tender lymph nodes, or headache) for ≥ 1 month. For all those reported to be *unwell* *with fatigue* (n=3425), a random sample of unwell individuals *without fatigue* (n=2134), and a random sample of *well* individuals (n=3113) were then asked to complete a detailed (~ 20 minute) telephone interview that collected demographic, economic, and medical information (Table S1). Of those asked, 2438 *unwell with fatigue*, 1429 *unwell without fatigue*, and 1756 *well* individuals completed the detailed interview. Based on their responses, we classified those who met criteria of the 1994 CFS case definition [1] as *CFS-like* (they reported fatigue lasting ≥6 months that was not alleviated by rest, that caused substantial reduction in occupational, educational, social or personal activities, and that was accompanied by at least four of the CFS-defining symptoms and they reported no exclusionary medical or psychiatric condition). We classified those who reported fatigue, cognitive impairment, unrefreshing sleep, muscle pain, joint pain, sore throat, tender nodes or headache but were not CFS-like as *unwell* (with or without fatigue); those who reported none of these were classified as *well*.

We invited all 469 classified as *CFS-like* illness, 505 randomly selected *chronically unwell* participants (unwellness for at least 6 months), 160 *prolonged unwell* (unwellness lasting between 1 and 6 months), and 481 well to participate in a 1-day clinical examination. The *prolonged unwell* and *well* were matched to the CFS-like group on age, sex, and race.

| **Table S1. Subject Classifications at Phone Screening and Clinical Evaluation** | | |  |
| --- | --- | --- | --- |
| **Initial Telephone Screen** | | |  |
| *Adult in Household with Symptom* ≥ *1 month* | | | Count |
| Contacted Households | | | 11,247 |
| Participating Households | | | 10,837 |
| **Screened Individuals in Participating Households** | | | 19,381 |
| **Resulting Classification** | | |  |
|  | Well | | 10,834 |
|  | Unwell, Not Fatigued | | 5,122 |
|  | Unwell, Fatigued | | 3,425 |
| **Detailed Interview** | | |  |
| *Identify CFS-like cases using 1994 case definition* | | |  |
| **Invited** | |  |  |
|  | Well | | 3,113 |
|  | Unwell, Not Fatigued | | 2,134 |
|  | Unwell, Fatigued | | 3,425 |
| **Participated** | | |  |
|  | Well | | 1,756 |
|  | Unwell, Not Fatigued | | 1,429 |
|  | Unwell, Fatigued | | 2,438 |
| **Resulting Classification** | | |  |
|  | Medical or Psychiatric Explanation for Illness | | 1609 |
|  | No Explanation for Illness | | 4,014 |
|  |  | Well | 1,329 |
|  |  | Prolonged Unwell (1 to 6 months) | 453 |
|  |  | Chronically Unwell (>6 months) | 1,763 |
|  |  | CFS-Like | 469 |
| **Clinical Evaluation** | | |  |
| *Diagnose CFS cases using 1994 case definition* | | |  |
| **Invited** | |  |  |
|  | Well | | 481 |
|  | Prolonged Unwell (≥ 6 months unwell) | | 160 |
|  | Chronically unwell (>6 months) | | 505 |
|  | CFS-Like | | 469 |
| **Participated** | | |  |
|  | Well | | 163 |
|  | Prolonged Unwell (≥ 6 months unwell) | | 60 |
|  | Chronically unwell (>6 months) | | 268 |
|  | CFS-like | | 292 |
| **Resulting Classification*** | | |  |
|  | Medical or Psychiatric Explanation for Illness | | 280 |
|  | No Explanation for Illness | | 500 |
|  |  | Not Fatigued (NF) | 124 |
|  |  | Insufficient Symptoms or Fatigue for CFS (ISF) | 264 |
|  |  | Chronic Fatigue Syndrome (CFS) | 113 |
| *Two participants could not be classified due to incomplete lab results and one additional participant, diagnosed with CFS, was excluded from the economic analysis because the necessary questionnaires were not completed. | | | |

Details on Estimating Sampling Weights

It was necessary to construct sampling weights for the economic analyses because the design of the study resulted in a large portion of individuals for whom sampling probabilities were not known. An especially large number of the individuals in the “not fatigued” (NF) group lacked probability weights.

The supplemental material provided details in the estimation strategy for the weights. Section A.1 describes the analytic sample, explains the sampling weights used in the estimation, and describes the reference population that corresponds to these weights.

**A.1 Analytic sample and sampling weights**

This study is based on analysis of completed clinical evaluations for a sample of individuals aged 18 through 59 in three Georgia sampling strata:

- A metropolitan stratum comprising metropolitan Atlanta (Fulton and DeKalb Counties);
- An urban stratum comprising two smaller cities, Macon and Warner Robins (with respective populations of 300,000 and 38,000), and
- A rural stratum consisting of rural areas in ten counties surrounding Macon and Warner Robins.

Analysis of these data is complicated by the fact that we do not know the sampling weights for part of the analytic sample. To address this, we created pseudo-weights based on a stylized characterization of the sampling process for this part of the sample. We also combined the samples for the three strata in order to provide more precise estimates of CFS effects.

The rest of this section describes:

- The way in which the sample was drawn (Section A.1.1)
- The development of the pseudo sampling weights (Section A.1.2), and
- The reference population for the combined three-strata sample (Section A.1.3).

**A.1.1 Sampling Procedures**

The sampling procedure within each stratum involved three stages – an initial telephone screening interview, a detailed telephone interview for a sample of those who completed the screening interview, and clinical evaluations for a sample of those who completed the detailed interview. There is considerable variation in the sampling rates for detailed interviews within each of the three sampling strata. These reflect adjustments for non-response and interrupted telephone service as well as differential sampling rates based on screener interview reports of fatigue or other symptoms associated with CFS.

Some individuals who completed the detailed interviews were excluded from the clinical evaluation sample. Individuals were excluded because of age (in a very few cases) and more commonly because of medical or psychiatric conditions that would preclude a diagnosis of CFS. Excluded individuals were not selected for clinical evaluations and therefore these cases lack data on employment, utilization, and so forth that were collected during the clinical visits.

Individuals who completed the detailed telephone interview and were not excluded were classified into three categories based on application of the Fukuda criteria to the results of the detailed interview: CFS-like, Chronically Unwell, or Well. A sample of non-excluded individuals who completed the detailed interview was selected for clinical evaluations. Determination of CFS was based on the clinical evaluation. Data for most of the outcome variables of interest were also collected during these clinical visits.

Clinical evaluations were used to identify additional excluded cases and classify individuals as CFS or non-CFS based on the Wagner criteria^1^ (i.e. Empirical CFS case definition), with the non-CFS category subdivided into Insufficient Symptoms/Fatigue (ISF) and Not Fatigued (NF) categories. The sample with completed clinical evaluations comprises 500 individuals (after dropping cases with exclusionary conditions). The Fukuda classification from the detailed telephone interview and Wagner Classification from the clinical evaluation are correlated but far from identical. A substantial proportion of CFS-like individuals had Wagner classifications of ISF rather than CFS. Conversely, CFS cases were found in all three of the Fukuda classifications; though the proportion with a Fukuda classification of Well is probably small (percentages in Exhibit 1 refer to the percentage in the sample and are not estimates of population proportions).

| **Exhibit A.1: Sample for Fukuda and Wagner Classifications (Omitting Excluded Cases)** | | | | |
| --- | --- | --- | --- | --- |
|  | **Wagner** | | |  |
| **Fukuda** | **Not Fatigued (NF)**  Number  (Percent of Row) | **Insufficient Symptoms/Fatigue (ISF)**  Number  (Percent of Row) | **Chronic Fatigue Syndrome (CFS)**  Number  (Percent of Row) | **Total**  Number  (Percent of Row) |
| **CFS-like** | 0  (0 %) | 66  (44%) | 83  (56%) | 149  (100%) |
| **Chronic Unwell** | 26  (15%) | 126  (70%) | 26  (15%) | 178  (100%) |
| **Well** | 98  (57%) | 72  (42%) | 3  (2%) | 173  (100%) |
| **Total** | 124  (25%) | 264  (53%) | 112  (22%) | 500  (100%) |

Note: percentages are sample percentages and are not weighted to reflect sampling rates. In addition, percentages may not add to 100 because of rounding.

All CFS-like individuals and a sample of Chronically Unwell individuals were selected for clinical evaluations (though many of those selected did not complete the interviews). These samples contain all but three of the CFS cases in the sample, as well as somewhat more than half of the non-CFS cases in the sample. After adjusting for non-response, we can treat these as probability samples with known sampling rates. In contrast the clinical evaluation sample of Well individuals was selected to match the sample of CFS-like individuals who completed their clinical evaluations. Cases from the Well sample were matched to these with respect to sampling stratum (Metropolitan, Urban, or Rural), sex, race (Black or non-Black), ethnicity (Hispanic/non-Hispanic), and age, the match criteria being relaxed as necessary to ensure at least one match. We know the sampling rates for all individuals who completed the detailed interview and the clinical evaluation sampling probabilities for those with detailed interview classifications of CFS-like or Chronically Unwell; we do not have the clinical evaluation sampling probabilities those with detailed interview classifications of Well.

**A.1.2 Pseudo Sampling Weights for the Sample of Completed Clinical Evaluations**

We have not been able to devise any reasonable approach to estimating CFS effects that do not require information on the sampling rates for the Well clinical evaluation sample. We cannot determine the actual sampling probabilities for this group. Matching was carried out without replacement as CFS-like cases completed their clinical evaluations, and criteria were relaxed when no match was available. We chose instead to try to develop pseudo-weights by using an analysis of the prevalence of CFS-like and Well cases among people who completed the detailed telephone interview to approximate the selection rates for this group.

We have response rate adjusted sampling weights for every completed clinical evaluation of individuals with Fukuda classifications from the detailed telephone interview of CFS-like or Chronic Unwell.^2^ We do not have sampling weights for completed clinical interviews of individuals with Fukuda classifications of “Well”. We impute pseudo weights for this group in each stratum based their detailed interview weights and a rough characterization of their response-adjusted clinical evaluation sampling rates. We then rescaled the weights to produce weights for the combined strata.

The pseudo-weights for the entire sample of completed clinical evaluations are given by:

(1) $w_{ij}=\left\{\begin{matrix} \left(ws_{j}\right)\left(CLIN\_EVAL\_WEIGHT_{ij}\right)\text{ } & \begin{matrix} \text{if Fukuda is CFS-like } \\ \text{or Chronic Unwell} \end{matrix} \\ \left(\text{ws}_{\text{j}}\right)\left(\text{wn}_{\text{j}}\right)\left(\text{wr}_{\text{ij}}\right)\left(\text{wp}_{\text{ij}}\right)\left(DET\_INT\_WEIGHT_{ij}\right) & \text{if Fukuda is Well } \end{matrix}\right.$

where

w_ij_ = the pseudo-weight for the i^th^ completed clinical evaluation in the j^th^ stratum;

CLIN_EVAL_WEIGHT_ij_ = the value of response-adjusted sampling weight for the i^th^

completed clinical evaluation in the j^th^ stratum, which is available for each completed clinical evaluation of cases with a detailed interview Fukuda classification of CFS-like or Chronic Unwell;

w_sj_ = the value of the stratum weight for the j^th^ stratum, defined below;

w_nj_ = the value of the stratum normalization for the j^th^ stratum, defined below;

wr^ij^ = the value of the non-response adjustment for the i^th^ completed clinical evaluation in the j^th^ stratum with a Fukuda classification of Well, defined below;

wp^ij^ = the value of the relative frequency adjustment for the i^th^ completed clinical evaluation in the j^th^ stratum with a Fukuda classification of Well, defined below; and

DET_INT_WEIGHT_ij_ = the value of the sampling weight for the i^th^ completed detailed interview in the j^th^ stratum with a Fukuda classification of Well.

The rest of this section defines the factors used to calculate w_ij_ for the Well sample.

The WP Factor. The sampling weight for the sample of clinical evaluations from individuals with a detailed interview Fukuda classification of Well is equal to the individual’s response-adjusted detailed interview sampling weight (DET_INT_WEIGHT) divided by the probability that the individual was selected for a clinical evaluation. The wp term reflects this inverse probability. As already indicated, the Well sample was selected to match the CFS-like clinical interview sample in terms of sampling stratum, sex, race, ethnicity, and age. Given this, it seems reasonable to approximate the expected sampling rate for people with given matching variable values in the Well sample by the ratio of the probability that such people have a classification of CFS-like to the probability that they have a classification of Well. The inverse of this rate (wp) is then given by:

(2) $wp_{ij}=\frac{\pi\left(x_{ij}\right)}{1-\pi\left(x_{ij}\right)}$

where

π(x_ij_) = the proportion of Well and CFS-like individuals with matching characteristics, x_ij_, who are Well.

We estimated π(x_ij_) using the sample of 2,251 completed detailed telephone interviews that were not excluded due to age or medical or psychological factors and had a Fukuda classification of either “CFS-like” or “Well”. Our sample is too small to support estimates for each cell defined by the values of stratum, sex, race, Hispanic and three-year age categories. We therefore smoothed the function considerably, specifying the probability that a person in this sample was Well as a logistic function of stratum (with rural as the omitted category), four age categories (18-29, 30-39, 40-49, and 50-59, with 40-49 as the omitted category), sex, race, Hispanic/non-Hispanic, and interactions of race with the two stratum dummies:

(3)

The estimated coefficients are shown in Exhibit A.2. The largest effects were associated with being male, being in the youngest age category (18-29), and being Black (in the Rural stratum), all of which were associated with a substantially higher probability of being Well.

| **Exhibit A.2: Estimated Logit for the WP Factor ^a^** | | | |
| --- | --- | --- | --- |
|  | **Estimate** | **Std. Err.** | **p-value** |
| **Intercept** | 0.3556 | 0.1186 | 0.003 |
| **Male** | 1.3563 | 0.1274 | <0.001 |
| **Black** | 1.0301 | 0.2049 | <0.001 |
| **Hispanic** | 0.2553 | 0.3222 | 0.428 |
| **Age 18-29** | 0.7986 | 0.1744 | <0.001 |
| **Age 30-39** | 0.3623 | 0.1507 | 0.016 |
| **Age 50-59** | -0.0753 | 0.1354 | 0.578 |
| **Metro Stratum** | 0.9483 | 0.1916 | <0.001 |
| **Urban Stratum** | 0.0351 | 0.1411 | 0.803 |
| **Metro*Black** | -1.2191 | 0.3399 | <0.001 |
| **Urban*Black** | -0.6331 | 0.2852 | 0.026 |
| n= 2251 | Mean p = 0.79 | Pseudo-R^2^ = 0.095 |  |
| Likelihood Ratio χ^2^= 219.4017 (10), p <0.0001 | | |  |

a) Logit for the probability that an individual is Well as a function of matching factors in the sample of completed detailed interviews with Fukuda classifications of Well or CFS-like.

As the pseudo-R2 indicates (and as would be expected), the model was not very effective in predicting Well status. The pseudo-R^2^ is computed as: , where y_i_ is one if the individual is well and 0 otherwise. However, our concern is not with predicting Well status, but with the variation in the predicted probabilities among the Well sample. These vary substantially, running from 0.57 to 0.98 for the Well sample, with a mean of 0.81 and an inter-quartile range of 0.76 to 0.90 (Exhibit A.3).

| **Exhibit A.3: Distribution of Predicted Probabilities for the Well Sample (n=1782)** | | | | | | | |
| --- | --- | --- | --- | --- | --- | --- | --- |
| **Mean** | **Percentiles** | | | | | | |
|  | **Min.** | **5%** | **25%** | **50%** | **75%** | **95%** | **Max.** |
| 0.81 | 0.57 | 0.58 | 0.76 | 0.84 | 0.90 | 0.96 | 0.98 |

The values of the wp_ij_ and their asymptotic errors of estimate are given by:

(4)

The variation in predicted probabilities produced a corresponding variation in wp values (Exhibit

A.4). The wp weighting factors ran from 1.3 to 44.5, with a mean of 7.7. Fortunately, extreme weights were relatively rare. Three fourths of the sample had weights less than 9, and 95% had weights less than 22.3.

| **Exhibit A.4: Distribution of the WP Factor for the Well Sample (n=1782)** | | | | | | | |
| --- | --- | --- | --- | --- | --- | --- | --- |
| **Mean** | **Percentiles** | | | | | | |
|  | **Min.** | **5%** | **25%** | **50%** | **75%** | **95%** | **Max.** |
| 7.74 | 1.32 | 1.37 | 3.16 | 5.32 | 8.88 | 22.29 | 44.51 |

To assess the error of estimate of the estimated weights, we computed the coefficients of variation for individuals with detailed interview classifications of Well. As shown in Exhibit A.5, these were generally small; the mean was 0.04 and 95% were less than 0.10.

| **Exhibit A.5: Distribution of The Estimation Coefficient of Variation for the WP Factors in the Well Sample (n=1782)** | | | | | | | |
| --- | --- | --- | --- | --- | --- | --- | --- |
| **Mean** | **Percentiles** | | | | | | |
|  | **Min.** | **5%** | **25%** | **50%** | **75%** | **95%** | **Max.** |
| 0.04 | 0.01 | 0.01 | 0.0.02 | 0.03 | 0.07 | 0.10 | 0.20 |

The WR factor. This is to adjust for non-response among those sampled for clinical evaluations.

Response rate adjustments for the rest of the clinical evaluation sample were based on variations in response rates associated with stratum, age, sex, and race. Rather than trying to develop an adjustment for the attempted clinical evaluations of Well cases, we used the response rate adjustments for the CFS-like clinical evaluation that was matched to the Well clinical evaluation:

(5) $wr_{ij}=NONEVAL\_ADJ\left(\text{LINKID}_{\text{ij}}\right)$

where

wr_ij_ = the value of the non-response adjustment for the i^th^ completed clinical evaluation in the j^th^ stratum with a Fukuda classification of Well;

LINKID_ij_ = the ID for the matched CFS-like case; and NONEVAL_ADJ is the value of this adjustment for the matching case.

The WN Factor. This is a normalization factor that is constructed so that, for each of the three strata, the sum of the pseudo-weights for Well sample clinical evaluations equals the sum of the detailed interview weights for the Well sample after exclusions (i.e., for the sample from which the clinical evaluation Well sample was drawn). Accordingly, wn_j_ is defined by:

(6) $\left(\text{wn}_{\text{j}}\right)=\frac{\sum_{i=1}^{N_{j}} \left(DI_{ij}\right)\left(1-EXDI_{ij}\right)\left(FWELL_{ij}\right)\left(DET\_INT\_WEIGHT_{ij}\right)}{\sum_{i=1}^{N_{j}} \left(\text{CE}_{\text{ij}}\right)\left(FWELL_{ij}\right)\left(\text{wr}_{\text{ij}}\right)\left(\text{wp}_{\text{ij}}\right)\left(DET\_INT\_WEIGHT_{ij}\right)}$

where the sums are over all cases in a stratum and

DI_ij_ = 1 if the i^th^ person in the j^th^ stratum has a completed detailed telephone interview and otherwise = 0;

EXDI_ij_ = 1 if the i^th^ person in the j^th^ stratum has either an age exclusion or a medical or psychiatric exclusion based on the detailed telephone interview, and otherwise = 0;

CE = 1 if clinical evaluation complete, else = 0; and

FWELL_ij_ = 1 if the i^th^ completed clinical evaluation in the j^th^ stratum had a Fukuda classification from the detailed telephone interview of “Well”.

The WS Factor. We estimated effects using the combined sample from the three strata. The WS

factor reflects the relative weight given to each stratum in this combined sample. One approach to this would be to weight the strata in proportion to the stratum populations. We rejected this on two grounds. First, the strata were constructed to include several different types of environments among places with reasonably easy access to clinical evaluation sites. Estimates specific to this somewhat artificial population have no obvious intrinsic interest. Rather, they are of interest to the extent that we believe that they are indicative of results for larger populations and not heavily influenced by the specific stratum composition of the sample. Furthermore, such estimates will be dominated by the Metropolitan stratum, which accounted for more than 85% of the sampled population. Because of this, we chose instead to weight each stratum in proportion to its number of clinical evaluations. This seemed likely to provide the most precise estimates, while still allowing us to take account of stratum in estimating effects.

The final weights for the analytic sample include some extremely high values. They have a mean

value of one, with a standard deviation of 3.4, and range from 0.03 to 56.03, though 95% are less than 16. This suggests that it might be useful to consider trimming the weights to the extent that large values are associated with non-response adjustments. (We would be more reluctant to trim weights that reflect differences in sampling rates associated with levels of reported fatigue, since these are directly related to the contrasts of interest.) We did not trim weights for this analysis.

| **Exhibit A.6: Distribution of Final Weights for the Analytic Sample (n=500)** | | | | | | | | |
| --- | --- | --- | --- | --- | --- | --- | --- | --- |
| **Mean** | **Std. Dev.** | **Percentiles** | | | | | | |
|  |  | **Min.** | **5%** | **25%** | **50%** | **75%** | **95%** | **Max.** |
| 1.00 | 3.45 | 0.029 | 0.055 | 0.107 | 0.303 | 0.795 | 2.92 | 56.035 |

**A.1.3 Composition of the Reference Population**

Since we weight each stratum in proportion to its number of completed clinical evaluations, the reference population for the analysis in this paper can be thought of as being a population in which

- 20% of the population is drawn from the Atlanta metropolitan area;
- 32% of the population is drawn from Macon and Warner Robins; and
- 48% of the population is drawn from the rural areas of counties surrounding Macon

and Warner Robins.

As described in the next section, estimated CFS effects are based on comparisons between outcomes of individuals with and without CFS. All of these comparisons exclude individuals who have medical or psychological conditions that preclude identification of CFS. As shown in Exhibit A.7, about 40% of the population in each stratum was excluded for these reasons.

| **Exhibit A.7: Estimated Percentage of the Population in Each Stratum That is Excluded for Medical or Psychological Reasons** | | |
| --- | --- | --- |
| **Metro Stratum** | **Urban Stratum** | **Rural Stratum** |
| 41.6% | 42.8% | 39.8% |

Non-CFS individuals are further classified as either NF or as ISF. In addition to comparing outcomes for individuals with CFS with outcomes for all non-CFS individuals, we also separately compare CFS outcomes with NF or ISF outcomes as well as comparing NF and ISF outcomes. Exhibit A.8 shows the proportions of individuals in the non-excluded reference population who fall into each of these groups.

Because percentages are computed relative to the non-excluded population, all of the prevalence rates are substantially higher than the rates in the population as a whole. The Wagner and Fukuda

classifications are clearly correlated, but far from identical. Only about 56% of individuals with a Fukuda classification of CFS-like have a Wagner Classification of CFS. This is more than offset by the fact that over 9% of the much larger group with a Fukuda classification of Chronic Unwell have a Wagner Classification of CFS. As a result, the percentage with a Wagner Classification of CFS is almost two thirds larger than the percentage with a Fukuda classification of CFS-like. Similarly, over 46% of those with a Fukuda classification of Well have a Wagner classification of ISF, so that the overall percentage with a Wagner Classification of ISF is more than four-fifths larger than the percentage with a Fukuda classification of Chronic Unwell.

| **Exhibit A.8: Estimated Percentage of the Non-Excluded Reference Population in Each Wagner/Fukuda Classification** | | | | |
| --- | --- | --- | --- | --- |
| **Wagner Classification** | **Fukuda Classification** | | | |
|  | **CFS-like** | **Chronic Unwell** | **Well** | **TOTAL** |
| **CFS** | 1.7 | 2.7 | 0.5 | 4.9 |
| **ISF** | 1.3 | 19.0 | 31.8 | 52.2 |
| **Not-Fatigued** | 0.0 | 6.9 | 36.0 | 42.9 |
| TOTAL | 3.0 | 28.7 | 68.4 | 100.0 |
