# Supplementary material for: The economic impact of chronic fatigue syndrome in Georgia: direct and indirect costs
Source: Cost Eff Resour Alloc. 2011 Jan 21;9:1. doi: 10.1186/1478-7547-9-1 (PMC3033815; doi:10.1186/1478-7547-9-1)
Supplement: Additional file 2 — Full model results for Tables 3 and 6. The file includes the tables for the full model results for Tables 3 and 6. The file also includes Table S4 for examining the impact of CFS one earnings from separate analyses controlling its interaction with the reported educational attainment and the employment status. [file 1478-7547-9-1-S2.DOC]

### Additional file 2 – full model results for Tables 3 and 6

**Table S2 - Estimated Parameters from the Annual Healthcare Expenditures Model**

Full generalized linear model (GLM) results

|  | Annual Healthcare Expenditures | | | | | |
| --- | --- | --- | --- | --- | --- | --- |
|  | Total | Inpatient Hospital | Provider Encounters | Rx Medications | OTC Medications | Other Health Costs |
| Independent Variables | Estimate  (Std Err) | Estimate  (Std Err) | Estimate  (Std Err) | Estimate  (Std Err) | Estimate  (Std Err) | Estimate  (Std Err) |
| CFS Indicator | 3285.54***  (1026.50) | 519.95  (582.17) | 1343.25**  (587.55) | 1241.13*  (694.19) | 204.01*  (111.16) | -1.83  (1.28) |
| ISF Indicator | 1058.27***  (352.68) | 71.61  (122.50) | 384.73  (250.49) | 317.41**  (131.90) | 46.83  (30.66) | 0.82  (1.37) |
| NF | Referent | Referent | Referent | Referent | Referent | Referent |
| Female | 1642.61***  (388.99) | 98.72  (128.77) | 977.85***  (259.63) | 695.56  (255.08) | 99.40***  (30.85) | 17.56  (14.45) |
| Male | Referent | Referent | Referent | Referent | Referent | Referent |
| Age 18 - 29 | Referent | Referent | Referent | Referent | Referent | Referent |
| Age 30 – 39 | 457.98  (597.05) | 724.15  (959.61) | 53.83  (396.74) | 274.66  (270.40) | 60.57  (58.14) | 0.18  (1.11) |
| Age 40 - 49 | 1148.83*  (635.65) | 2959.02  (3088.20) | 124.63  (383.89) | 940.24  (657.12) | 290.63  (173.24) | 12.13  (12.61) |
| Age 50 - 59 | 3933.01***  (378.86) | 11432.39  (8415.00) | 1276.20*  (656.91) | 2540.92*  (1529.50) | 222.16  (153.40) | 51.36  (53.48) |
| Non-Hispanic White | 1115.58***  (378.86) | -32.19  (115.29) | 594.16**  (264.72) | 237.47***  (78.62) | 2.96  (33.35) | -0.47  (0.66) |
| Non-white | Referent | Referent | Referent | Referent | Referent | Referent |
| Urban | 652.14  (430.40) | 418.87  (484.54) | 295.30  (257.83) | 37.71  (113.81) | 1.21  (24.91) | -0.66  (1.98) |
| Metro | -254.44  (458.27) | -56.11  (68.25) | -166.59  (347.31) | -50.35  (99.46) | 89.33  (71.48) | -2.25  (3.29) |
| Rural | Referent | Referent | Referent | Referent | Referent | Referent |
| * p < 0.1 ** p < 0.05 *** p < 0.01 | | | | | | |

**Table S3 - Estimated Effect of CFS on Employment Status and Earnings**

Logistic regression for the employment rate; generalized linear model for earnings

|  | Productivity | | | |
| --- | --- | --- | --- | --- |
|  | Probability of Any Work in Last 4 weeks (Reported Educational Attainment) | Probability of Any Work in Last 4 weeks (Imputed Educational Attainment) | Estimated Parameters of the Total 4-week Earnings Model (Reported Educational Attainment) | Estimated Parameters of the Total 4-week Earnings Model (Imputed Educational Attainment) |
| Independent Variables | OR  (Std Err) | OR  (Std Err) | Estimate  (Std Err) | Estimate  (Std Err) |
| CFS Indicator | 0.15***  (0.10) | 0.12***  (0.08) | -503.11  (392.52) | -658.30**  (298.83) |
| ISF Indicator | 0.14***  (0.08) | 0.14***  (0.08) | -1019.04  (654.78) | -1029.23  (665.62) |
| Age 18-29 | Referent | Referent | Referent | Referent |
| Age 30 - 39 | 1.90  (1.19) | 1.89  (1.17) | 2860.58***  (1013.40) | 2849.99***  (993.39) |
| Age 40-49 | 1.31  (0.69) | 1.29  (0.68) | 2697.16*  (1429.30) | 2721.37*  (1456.30) |
| Age 50-59 | 0.43*  (0.22) | 0.43*  (0.22) | 1836.42**  (764.72) | 1837.66**  (746.23) |
| Married Male | 11.42***  (8.38) | 11.97***  (8.83) | 515.24  (730.82) | 521.32  (712.56) |
| Unmarried Male | 0.63  (0.37) | 0.64  (0.37) | -1280.08  (1029.40) | -1291.42  (1021.30) |
| Unmarried Female | 2.91*  (1.63) | 2.95*  (1.66) | -184.82  (359.85) | -183.79  (357.64) |
| Non-Hispanic White | 3.43***  (1.59) | 3.41  (1.58) | 1270.51*  (484.58) | 1281.50*  (761.29) |
| Urban | 0.51  (0.22) | 0.50  (0.22) | -667.43  (484.58) | -681.69  (485.04) |
| Metro | 2.52  (1.71) | 2.38  (1.62) | 753.64  (589.04) | 726.29  (580.39) |
| BA and Post-grad education | 3.17**  (1.49) | 3.45**  (1.69) | 1336.30***  (412.31) | 1194.75***  (317.09) |
| Un-weighted n | 498 | 498 | 499 | 499 |
| * p < 0.1 ** p < 0.05 *** p < 0.01 | | | | |

***Table S4 - Impact of Fatigue on Earnings Adjusting for the Interaction with the employment and Educational Attainment Separately.***

| *Marginal Effect After GLM on Earnings (y+1),* (*Standard Errors in Parentheses)* | | | |
| --- | --- | --- | --- |
|  | Model with the interaction of reported education and fatiguing groups |  | Model with the interaction of reported education and fatiguing groups |
| CFS without BA or Post Graduate Degree | -1320.12***  (347.08) | CFS without the Employment | -573.77***  (40.76) |
| CFS with BA or Post Graduate Degree | -610.69  (544.01) | CFS with the Employment | -40.38  (84.71) |
| ISF without BA or Post Graduate Degree | -1036.88**  (517.29) | ISF without the Employment | -2243.20***  (214.01) |
| ISF with BA or Post Graduate Degree | -807.15*  (434.59) | ISF with the Employment | 20.55  (89.55) |
| NF without BA or Post Graduate Degree | -1732.20***  (557.39) | NF without the Employment | -610.47***  (44.03) |
| NF with BA or Post Graduate Degree | Referent | NF with the Employment | Referent |
| * p < 0.1 ** p < 0.05 *** p < 0.01 | | | |
